# Editorial: Reducing the Burden of Age-Related Disease in Relation to Osteoporosis, Sarcopenia and Osteosarcopenia

**DOI:** 10.3389/fmed.2022.882140

**Published:** 2022-04-25

**Authors:** Ozra Tabatabaei-Malazy, Ali Tootee, Ramin Heshmat, Afshin Ostovar, An Pan, Arshed Ali Quyyumi, Farshad Farzadfar, Bagher Larijani

**Affiliations:** ^1^Non-Communicable Diseases Research Center, Endocrinology and Metabolism Population Sciences Institute, Tehran University of Medical Sciences, Tehran, Iran; ^2^Cell Therapy and Regenerative Medicine Research Center, Endocrinology and Metabolism Molecular-Cellular Sciences Institute, Tehran University of Medical Sciences, Tehran, Iran; ^3^Chronic Diseases Research Center, Endocrinology and Metabolism Population Sciences Institute, Tehran University of Medical Sciences, Tehran, Iran; ^4^Osteoporosis Research Center, Endocrinology and Metabolism Clinical Science Institute, Tehran University of Medical Sciences, Tehran, Iran; ^5^Ministry of Education Key Laboratory of Environment and Health, Department of Epidemiology and Biostatistics, School of Public Health, Tongji Medical College, Huazhong University of Science and Technology, Wuhan, China; ^6^Emory Clinical Cardiovascular Research Institute, Emory University, Atlanta, GA, United States; ^7^Endocrinology and Metabolism Research Center, Endocrinology and Metabolism Clinical Sciences Institute, Tehran University of Medical Sciences, Tehran, Iran

**Keywords:** osteoporosis, sarcopenia, osteosarcopenia, aging, physical frailty, bonehealth, muscle, burden

This Research Topic collection entitled “*Reducing the Burden of Age-Related Disease in Relation to Osteoporosis, Sarcopenia and Osteosarcopenia*,” developed by authors from various countries, aims to investigate different strategies for screening, diagnosis, and management of sarcopenia and osteoporosis as major public health threats to aging populations. The articles published in this Research Topic introduce novel imaging and laboratory approaches for screening and early detection of osteoporosis and sarcopenia, and propose effective prevention and treatment strategies. They also provide evidence as to how newly emerged nutrition, physical activity, and medication approaches can effectively prevent devastating complications and consequences of sarcopenia and osteoporosis.

Sarcopenia is associated with a high risk for joint pain, functional dependence, institutionalization, high health costs, and mortality. Considering that a high prevalence of sarcopenia in elderly populations often remains undiagnosed, development of novel screening techniques for its detection is of crucial importance. Although, there exist various screening tools in the clinical setting, most of them have disadvantages such as cost, length of the procedure, and low diagnostic accuracy. One of the most widely used techniques for screening sarcopenia is bioelectrical impedance analysis (BIA) which is a relatively simple, inexpensive, fast, non-invasive, and reliable technique. In the first article of this Research Topic, the authors propose a phase angle (PA) cut-off point for screening sarcopenia in a population of elderly Mexican people, and conclude that PA can be an effective indicator for timely detecting sarcopenia and frailty (Rosas-Carrasco et al.). In the second article, Shafiee et al. report development of a simple, non-invasive, cost-effective, and practical tool called SarSA-Mod for screening sarcopenia in both genders. They propose that considering the fact that SarSA-Mod is an easy-to-calculate diagnostic procedure with simple variables, it can be considered as an effective screening model for early detection of sarcopenia in a primary care setting.

Sarcopenia is associated with discomfort, disability, and pain in articular areas such as the shoulders. The third article investigates the possible relationship between sarcopenia and rotator cuff tendon diseases (Han et al.). The authors observe that although sarcopenia is associated with shoulder pain, it does not lead to serious rotator cuff injuries such as tendon tears. Exercise improves muscle strength, physical performance, and mental states such as cognition and mood. In the fourth article, the authors conduct a systematic review to examine the effects of exercise on muscle strength, body composition, and physical performance in older adults (Zhang et al.). In their meta-analysis, they report that exercise has positive effects on muscle strength, physical performance, and skeletal muscle mass in sarcopenia. Nonetheless, they contend that exercise does not alter body composition (e.g., fat mass, lean mass, and fat-free mass) in elderly people with sarcopenia.

In the fifth article, Azzolino et al. highlight nutrition and physical exercise as two important environmental factors which can effectively promote musculoskeletal health. In their review article, they briefly describe body composition changes across the lifespan and propose several nutrition and exercise strategies aiming at promotion of musculoskeletal health and delaying the aging process. This is in agreement with the sixth article which evaluates the impact of community-based dual-task exercise on muscle strength and physical function in sarcopenia. They analyze the effects of dual-task exercise on cognition, frailty, falls, social isolation, and perceived health in the elderly (Merchant et al.). Their results demonstrates that a dual-task exercise program is significantly effective in improving gait, speed, physical performance, handgrip strength, perceived health, and cognition, and reduces frailty and falls.

The authors of the seventh article contend that hitherto the link between sarcopenia and food-based inflammatory potential of the diet (FIPD), which demonstrates pro-inflammatory quality of the diet, has remained unexplored, and claim that their study has shed light on this topic (Bagheri et al.). Closely examining the association between FIPD and sarcopenia (and its components), they maintain that a greater FIPD score is positively linked with sarcopenia components, and propose that lower consumption of foods with a greater pro-inflammatory capacity, and higher intake of foods with anti-inflammatory features, may have a protective effect against sarcopenia.

The author of the eighth article describes HEPAS (healthy eating, physical activity, and sleep hygiene) as a multidisciplinary approach which can promote physical and mental health and wellbeing of individuals with neuropsychiatric diseases (Briguglio). In this opinion article, the author elaborates, in details, how HEPAS can be exploited to prevent sarcopenia, and comments that HEPAS can improve grip, muscle strength, and skeletal muscle mass in the elderly. The author ultimately concludes that this lifestyle modification strategy can be of great value for the promotion of the health of both community-dwelling and institutionalized elderly people.

Sarcopenia is associated with several extra-muscular complications such as mental disorders, cardiovascular diseases, and hypertension. Cardiovascular (CVD) complications of sarcopenia can be quite devastating, and can be detected by electrocardiography (ECG). In the ninth article, the authors report that in their elderly population with sarcopenia, ECG abnormality and the risk of cardiovascular involvement was significantly increased (Heshmat et al.). In the tenth article, the authors point out that although obesity is documented to be associated with hypertension, the link between sarcopenia (and sarcopenic obesity) and hypertension remains mostly obscure (Pasdar et al.). In a cross-sectional study conducted on 4,021 cases from Ravansar, Iran, they report that obesity was associated with hypertension, whereas sarcopenia and sarcopenic obesity had no such relationship with hypertension.

In the eleventh article, the authors comment that although cadmium (Cd) is linked to osteoporosis and osteopenia, there have found conflicting reports about this relationship (Li et al.). In their meta-analysis, they assert that while urine Cd concentration may be related to increased risk of osteoporosis and osteopenia, blood Cd concentrations have no such relationship. In the end, they propose that measurement of urine Cd concentration can provide a reliable assessment tool for screening and diagnosis of osteoporosis and osteopenia, whereas blood Cd concentrations are of no such diagnostic value.

The twelfth article reports the findings of a prospective multi-institutional randomized controlled study which aims to investigate whether zoledronate prevents loss of bone mineral density (BMD) after discontinuation of denosumab (Lee et al.). They conclude that although a single dose of zoledronate may be helpful, individuals' response to sequential therapy can widely vary based on the baseline fracture risk, bone turnover rate, and duration of denosumab treatment amongst several other factors.

To conclude, appreciating the high health burden of sarcopenia and osteoporosis, all articles of this Research Topic collection propose novel techniques and strategies for screening, diagnosis, prevention, and management of these two silent and devastating adverse health conditions of the elderly. This topic Research Topic can be considered as a great contribution to the body of scientific evidence in the field and the articles illuminate different obscure aspects of screening, diagnosis, and management of osteosarcopenia. Moreover, they highlight current research gaps and elucidate the path for future research on the topic, and put forward practical strategies to address scientific shortcomings and insufficiencies.

## Author Contributions

All authors listed have made a substantial, direct, and intellectual contribution to the work and approved it for publication.

## Conflict of Interest

The authors declare that the research was conducted in the absence of any commercial or financial relationships that could be construed as a potential conflict of interest.

## Publisher's Note

All claims expressed in this article are solely those of the authors and do not necessarily represent those of their affiliated organizations, or those of the publisher, the editors and the reviewers. Any product that may be evaluated in this article, or claim that may be made by its manufacturer, is not guaranteed or endorsed by the publisher.

